# A Fish Assemblage from the Middle Eocene from Libya (Dur At-Talah) and the Earliest Record of Modern African Fish Genera

**DOI:** 10.1371/journal.pone.0144358

**Published:** 2015-12-16

**Authors:** Olga Otero, Aurélie Pinton, Henri Cappetta, Sylvain Adnet, Xavier Valentin, Mustapha Salem, Jean-Jacques Jaeger

**Affiliations:** 1 Institut de Paléoprimatologie, Paléontologie Humaine: Évolution et Paléoenvironnents (iPHEP) - UMR CNRS 7262, Université de Poitiers, Poitiers, France; 2 Institut des Sciences de l'Évolution de Montpellier (ISE-m) - UMR5554, Université Montpellier 2, Montpellier, France; 3 Geology Department, University of Al-Fateh, Tripoli, Libya; University of Oxford, UNITED KINGDOM

## Abstract

In the early nineteen sixties, Arambourg and Magnier found some freshwater fish (i.e., *Polypterus* sp., Siluriformes indet. and *Lates* sp.) mixed with marine members in an Eocene vertebrate assemblage at Gebel Coquin, in the southern Libyan Desert. This locality, aged ca 37–39Ma and now known under the name of Dur At-Talah, has been recently excavated. A new fish assemblage, mostly composed of teeth, was collected by the Mission Paléontologique Franco-Libyenne. In this paper, we describe freshwater fish members including a dipnoan (*Protopterus* sp.), and several actinopterygians: bichir (*Polypterus* sp.), aba fish (*Gymnarchus* sp.), several catfishes (*Chrysichthys* sp. and a mochokid indet.), several characiforms (including the tiger fish *Hydrocynus* sp., and one or two alestin-like fish), and perciforms (including the snake-head fish *Parachanna* sp. and at least one cichlid). Together with the fossiliferous outcrops at Birket Qarun in Egypt, the Libyan site at Dur At-Talah reduces a 10-Ma chronological gap in the fossil record of African freshwater fish. Their fish assemblages overlap in their composition and thus constitute a rather homogenous, original and significant amount of new elements regarding the Paleogene African ichthyofauna. This supports the establishment of the modern African freshwater fish fauna during this time period because these sites mostly contain the earliest members known in modern genera.

## Introduction

The Paleogene time period has been long considered critical for the fossil record of fish as we face extensive chronological gaps and poor taxonomic determination of the existing fossils. Increasing knowledge of the Eocene and Oligocene, thus, appears necessary to document the evolutionary history of fishes in continental waters. This was notably achieved with a few works in the last decade dealing with the systematic revision of Paleogene fish taxa from Africa (e.g., [1,2,3]) and featuring the pattern of fish fossil record and its control [4]. In comparison, the discovery of new material is rather rare. For instance, in the last 10 years, a single Paleogene fossil fish assemblage was described from the Late Eocene historical outcrops of Egypt at the Fayum Depression [5,6]. Here, we describe the Paleogene fish assemblage from Dur At-Talah (Sirte Basin, Libya) recently collected by the Mission Paléontologique Franco-Libyenne, in the Mid-Late Eocene sedimentary deposits.

Dur At-Talah was dated between 38 and 39 Ma (upper Bartonian) on the basis of biochronology and magnetochronology [7], and the Birket Qarun assemblage was dated about 37 Ma (early Priabonian) based on biochronological arguments [8,9]. Since younger African sites with freshwater fish are dated to the Lower Oligocene, and older ones are from the Ypresian and Lutetian (see [10] for details), the Egyptian and Libyan sites at Birket Qarun and Dur At-Talah thus fall within an over 10 Ma chronological gap in the fossil record of African freshwater fish. Indeed, Birket Quarun assemblage description [6] constituted an original and significant amount of new elements about Late Eocene African freshwater fish fauna. And featuring the fish assemblage at Dur At-Talah allows to compare for the first time African fish faunas in the Late Eocene and discuss the similarities and differences existing in these sub-contemporary and geographically close basins in this time period.

In a previous study of the vertebrate assemblage at Gebel Coquin (a former name for Dur At-Talah), Arambourg and Magnier found freshwater fish (i.e., *Polypterus* sp., Siluriformes indet. and *Lates* sp.) mixed with marine members [11]. As expected, our assemblage, which mostly includes screening samples, provides a different, more diverse and complementary data set.

## Geological Context

**Fig 1:**
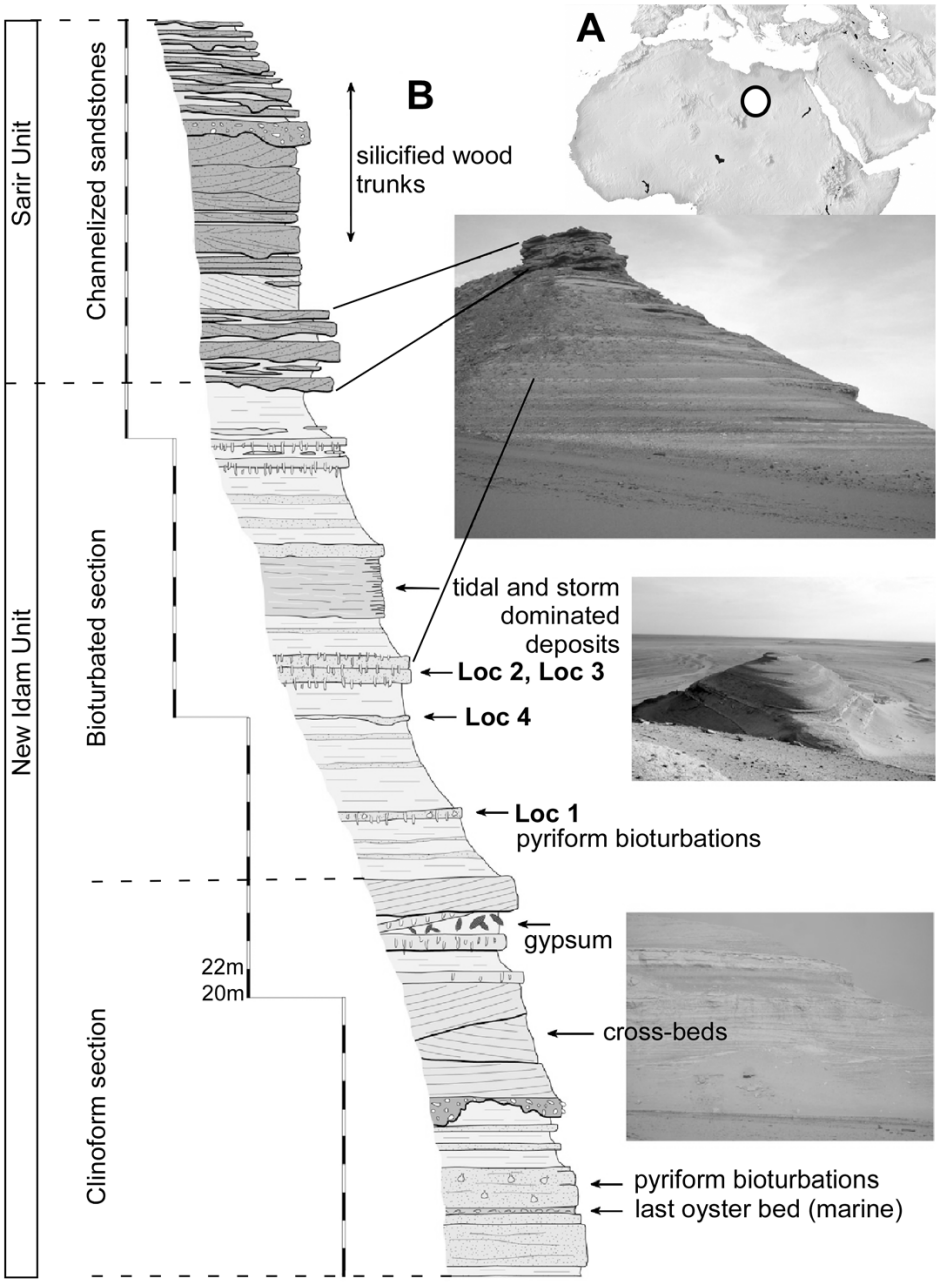
The fossiliferous site at Dur At-Talah. **A**, Location of the Dur At-Talah outcrop in Northern Africa, and, **B**, synthetic stratigraphic column of the Dur At-Talah series showing certain dominating facies and the relative position of the micro-vertebrate levels (from [7] modified). The Unit definition is from [17] and the section names follow [7], scale bar is 2 m. [planned for column width].

The fossil vertebrate area of Dur At-Talah, located in the Sirt Basin, southeastern Libya, has been known for over 50 years. This fossiliferous escarpment (Fig 1) was first explored during the second half of the last century notably by paleontologists under the leadership of C. Arambourg in the nineteen fifties and by a team of paleontologists led by R.J.G. Savage in the late nineteen sixties [12,11,13,14,15,16]. Field surveys were undertaken in 2008 and they are currently conducted within the framework of the Mission Paléontologique Franco-Libyenne. These renewed fieldwork efforts led to significant improvements of the fossil record at Dur At-Talah, with a new set of biochronological and biogeographical results.

The stratigraphy of the section consisted of three Units (Fig 1B) overlying basal marine late middle Eocene deposits. The lowermost Evaporite Unit and the Bioturbated Unit (Idam Unit of [16]) recognised by Jaeger et al. [7] were gathered in the New Idam Unit by Abouessa et al. [17]. The uppermost and nearly azoic sandstones called Channelized Sandstone Unit by Jaeger et al. [7] correspond to the Sarir Unit of Wright [16] and Abouessa et al. [17]. This series exhibits the succession of three distinct facies: a littoral marine one, a fluvio-deltaic one and a fluvial dominated one. The Bioturbated Unit delivered the freshwater fish fossil remains, collected by wet screening from centimetric fluviatile channels and overbanks deposits in which freshwater fishes are dominant over brackish water sharks and rays. However, over- and under-lying sediments associated with these small channel deposits yield only marine vertebrates. A coarse-grained fluvial deposit on the upper part of the section marks the final stage of a general marine Unit characterised by a mosaic of coastal facies in a mixed tidal/storm-dominated environment. Fossil mammals also concentrate in the same middle Unit, as well as in the lower one, but the faunal composition shows no marked difference between them, suggesting that they represent one single formation.

Additional paleontological data permit an estimation of the age by the interpolation of the geomagnetic data with the biochronological information obtained from the mammal association. Indeed, the whole series (Fig 1B) belongs to a single, long normal polarity and Jaeger et al. [7] retained three long normal polarities to which it might correspond: one within Chron C16 (C16n.2n about 36.3 to 35.7 Ma), another within C17 (C17n.1n, about 37.2 to 36.5 Ma old), and a third in C18 chron (C18n.1n between 39 and 38 Ma). Finally, the mammalian association and the long duration of the normal polarity recorded at these rodent-bearing layers of the fossiliferous Idam Unit (‘Bioturbated Unit’) support a correlation rather with Chron 18n.1n dated between 39 and 38 Ma (i.e., upper Bartonian) according to Jaeger et al. [7]. Further study of the marine shark assemblages collected in the marine levels that are interlayered between freshwater ones, might provide new elements that constrain the biochronological dating of the outcrops.

## Material

The surveys of the long escarpment of Dur At-Talah have led to the discovery of several microfossil concentrations in the western part of this escarpment, within the same sedimentary Unit. These fossiliferous lenses represent nearly synchronous localities according to magnetostratigraphic data. More than 300 kg of sediment (claystones) and up to 3 tons (for Locality 2) were collected at each locality for a total amount of about 6 tons. Intensive screen washings have yielded diverse assemblages of aquatic and terrestrial vertebrates (fishes, turtles, crocodiles, squamates), together with terrestrial mammals (such as rodents, bats, creodonts, marsupials, elephant shrews, hyraxes, and primates). The mesh of the screen is 1 mm. The fish material collected at Dur At-Talah is fragmentary and was mainly collected when screening sediment to sample micro-mammals during the last campaigns (2008–2010). Most of the material was collected at the locality Loc2 [7]. Depending on the fossils, the surfaces might be weathered or not. Moreover, fossil bones are scarce and fragmentary, whereas teeth largely dominate the fish assemblage, which might be possibly due to teeth resilience. This preservation is also probably partially related to the assumed fluvio-deltaic environment of relatively high energy, but also in direct relation to the screen-sampling method.

The research permit agreement was established between the University of Tripoli and the University of Poitiers for the Mission Paléontologique Franco-Libyenne, and covered a five year time period (2008–2012, renewed for 2013–2017), under the two principal investigators Mustapha Salem (University of Tripoli) and Michel Brunet (University of Poitiers). The samples were numbered by batch. The number refers to the fossiliferous site (DT for Dur At-Talah), the collection year, the group (O for “osseous fish”) and then the batch number (Table 1). The material is temporarily held at the University of Poitiers (France) but will eventually be transferred to its permanent home in the Paleontological Collections of the Department of Earth Science in the University of Tripoli (Libya): whilst waiting for correct logistical and access conditions, it is.

**Table 1:** Sample locality and content. For each sample, the number of elements is given (including fragments).

		DAT-2009-O-1	DAT-2009-O-2	DAT-2007-O-1	DAT-2008-O-1	DAT-2009-O-3	DAT-2009-O-4	DAT-2009-O-5
	Locality number	Loc. 1	Loc. 1	Loc. 2	Loc. 2	Loc. 2	Loc. 3	Loc. 4
*Protopterus* sp.	jaw	5	4	1	-	7	3	-
*Polypterus* sp.	scale	20	4	-	9	9	-	-
	pinnula	-	1	-	-	-	-	-
	jaw tooth	-	1	-	-	-	-	-
*Gymnarchus* sp.	jaw tooth	8	6	-	-	5	-	1?
*Egertonia* sp.	tooth patch	-	-	1	-	-	-	-
*Hydrocynus* sp.	jaw tooth	6	4	-	-	1	9	2
?Characiformes	bicuspidate tooth	-	-	-	-	-	2	3
and? Alestidae	multicuspidate tooth	-	-	-	-	-	-	6
Siluriformes	parieto-supraoccipital	2	-	-	-	-	-	-
	tooth	-	-	-	-	-	-	-
	vertebra	-	-	-	-	-	3	-
	fin spine	3	-	-	-	3	1	-
*Parachanna* sp.	dentary	1	-	-	-	-	1	-
cf. Cichlidae	pharyngeal jaw	-	-	-	-	-	2	-
	spine	-	-	-	-	1	-	-
teleost indet.	pharyngeal jaw	2	10	-	-	9	11	10
	tooth	2	2	-	-	-	1	-

## Systematic Palaeontology

Class SARCOPTERYGII

Order LEPIDOSIRENIFORMES

Family PROTOPTERIDAE

*PROTOPTERUS* (Owen, 1839) [18]

**Fig 2:**
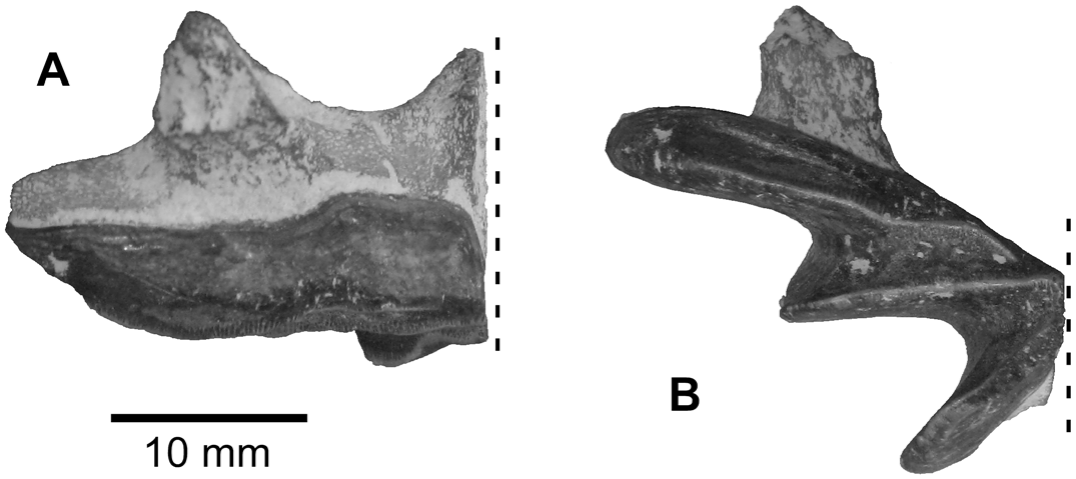
*Protopterus* sp. from the upper Bartonian deposits in Dur At-Talah, Libya. Left upper jaw (DT-2007-O-1) in, **A**, lingual, and, **B**, occlusal views. Dashed line corresponds to the symphysial plan in the front and horizontal sections, respectively. [planned for column width].

*PROTOPTERUS* sp. (Fig 2)

Lungfish remains consist of one large left upper jaw tooth plate preserved in connection with the jaw bone (Fig 2) and a dozen of smaller lower and upper jaws that are more or less fragmentary. Only one morphological type is clearly identified, suggesting that the material probably belongs to a single species. The tooth plate crests are ornamented by minute crenulations on both the upper and lower jaws. On the upper jaw, the three crests are separated by deep sulci (Fig 2A) and are of the same height (Fig 2B). There is a ridge along most of the inner flank of the third crest (Fig 2A). At the symphysis, the entopterygoid articulates with its counterpart through a striated articular surface which is oval in outline. When excluding the symphysal enlargement and the pterygopalate process, which both project dorsally, jaw bones are roughly as high as the tooth plates (Fig 2B). In occlusal view, the lower jaw is straighter than the upper. It counts three crests separated by deep sulci. The small lower jaws and jaw fragments do not permit a more precise description.

These jaws and jaw fragments are confidently attributed to an African lungfish, genus *Protopterus*, because of the presence of three crests only and of their rather gracile outline, which represents the most common condition in the genus [3]. Indeed, the other African lungfishes *Ceratodus* and *Lavocatodus* (fossils) exhibit more massive upper jaws [19,20]. When compared with modern and fossil species, the *Protopterus* from Dur At-Talah reaches too large a dimension to be related to an extant species, and it is too massive to be related to the Cretaceous *P*. *nigeriensis* [21]. Finally, the equal height of the three crests in the upper jaw avoids relating the lungfish from Dur At-Talah to *P*. *elongus*, a species described from the middle Eocene of Tilemsi, Mali [19] and identified in the early late Eocene from Egypt [6]. The Libyan material rather resembles a *Protopterus* with a large tooth plate from the Thanetian of the Morocco (Ouarzazate Basin), which was left *incertae sedis* [19]. As systematic attributions of African protopterids on the base of their jaw morphology remain weak [3], we place the Libyan material in *Protopterus sp*.

Class ACTINOPTERYGII

Order POLYPTERIFORMES

Family POLYPTERIDAE

*POLYPTERUS* Lacepède, 1803 [22]

**Fig 3:**
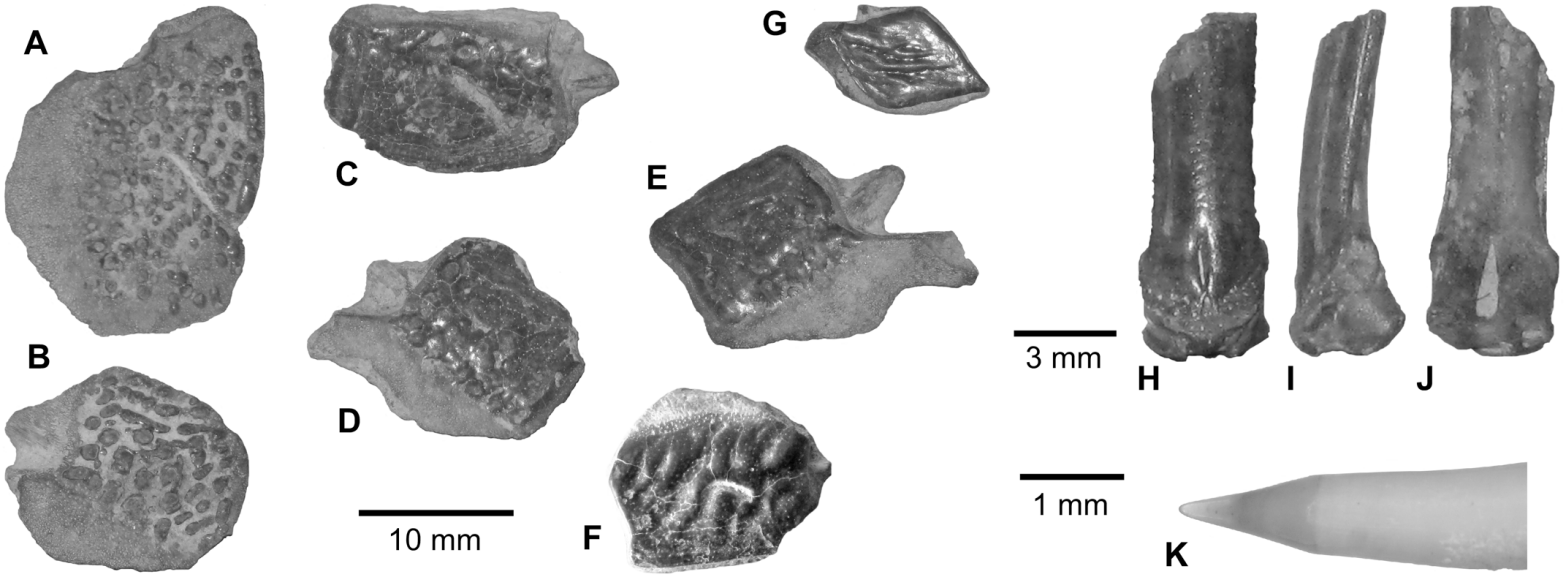
*Polypterus* sp. remains from the upper Bartonian deposits in Dur At-Talah, Libya. **A**–**E**, scales (DT-2009-O-2), **F**, scale (DT-2007-O-1), **G**, scale, and, **H**–**J**, pinnula (DT-2009-O-2), and, **K**, jaw tooth (DT-2009-O-2). [planned for page width].

*POLYPTERUS* sp. (Fig 3)

Skull roof bones, scales (Fig 3A–3G) together with one pinnula (Fig 3H–3J) and one tooth (Fig 3K) belong to polypteriform fishes. The pinnula is the spine that characteristically supports each dorsal finlet in these fish only. It is easily recognised by its articular head and the ganoid cover found on the anterior face (Fig 3H–3J). The ganoin, a peculiar type of enamel found in polypteriforms and recognised here, covers the outer surface of the scales and certain skull bones. The Dur At-Talah specimens exhibit various ornamented ganoid covering. There might be either more or less large ridges (Fig 3F and 3G), or no ridges and a rather smooth surface (Fig 3C), or the presence of tubercles (Fig 3D). Moreover, the covering might be continuous (Fig 3F and 3G) or lacunar (Fig 3C–3E) and might even result in a ganoid spot covering (Fig 3A and 3B). On the scales, the lateral line canal opens in a pore (Fig 3F) or in a gutter (Fig 3A and 3C) depending on the fossil specimen. The polypterid teeth exhibit a high tubular shape finished by a small pointed conical crown covered by enamel (Fig 3K).

Modern polypteriforms include the two genera *Polypterus* (bichir) and *Erpetoichthys* (rope fish). The first is largely distributed in African freshwaters, whereas the second is restricted to a short portion of the western African southern coast [23]. So far, all of the scales, skull bones and pinnules with a ganoid covering that have been collected in African Tertiary deposits have been attributed to *Polypterus*. This is notably the case of ganoid scales and of a tooth from the early late Eocene of Egypt [6]. In certain *Polypterus* species only, the canal opens in a pore on the lateral line scale. However, in these taxa, lateral line scales with a gutter also exist. Therefore, we attribute Dur At-Talah remains to an indeterminate *Polypterus* fish. Finally, today several species may co-exist in a single hydrographical basin, therefore we cannot speculate on the number of species belonging to the extinct community from Dur At-Talah.

Order OSTEOGLOSSIFORMES

Family GYMNARCHIDAE

*GYMNARCHUS* Cuvier, 1829 [24]

**Fig 4:**
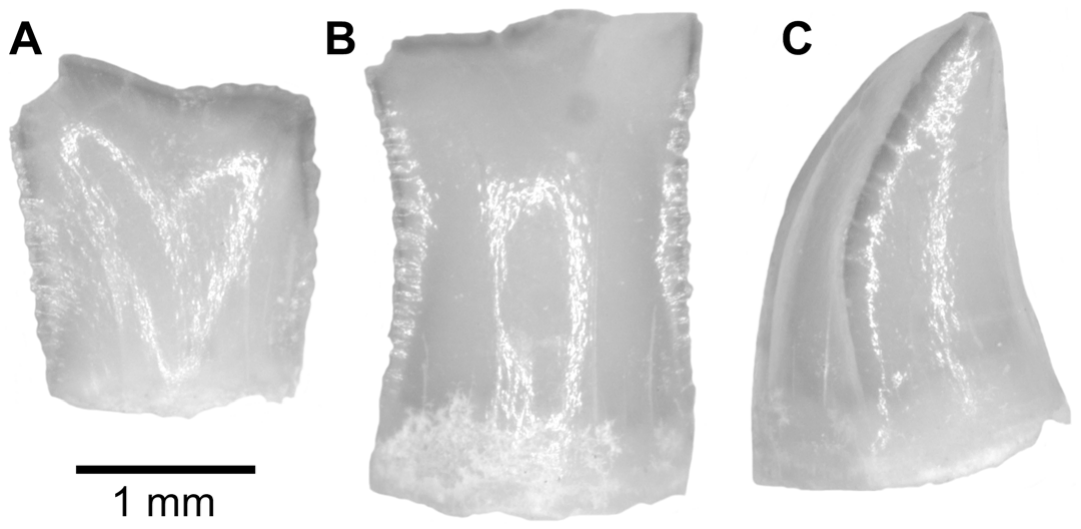
*Gymnarchus sp*. teeth from the upper Bartonian deposits in Dur At-Talah, Libya (DT-2009-O-5). In, **A**, lingual, **B**, labial, **C**, lateral views. [planned for column width].

*GYMNARCHUS* sp. (Fig 4)

*Gymnarchus* teeth have been collected in Dur At-Talah (Fig 4). The lingual face is quit flat, whereas the labial one is slightly convex. The cutting edge is finely serrated. A tubular cavity opens at the base of the tooth in a tiny hole. Modern *Gymnarchus* have teeth that vary in shape from incisiform at the symphysis to caniniform distally on the jaw. In Dur At-Talah, all *Gymnarchus* teeth are found disarticulated, and this range of shape variation is found in the assemblage (Fig 4).

In all of their characters, the teeth from Dur At-Talah are similar to the extant species *Gymnarchus niloticus* (the aba fish), the single living species in the genus and even in the family. We follow Murray et al. [6] who reported aba fish teeth from early late Eocene deposits of Egypt as *Gymnarchus* sp., and report the aba fish teeth from Dur At-Talah to the genus but not to the extant species. Both fossil and living aba fish inhabit African inland waters [25,10].

Order ELOPIFORMES

Family PHYLLODONTIDAE

*EGERTONIA* Cocchi, 1864 [26]

**Fig 5:**
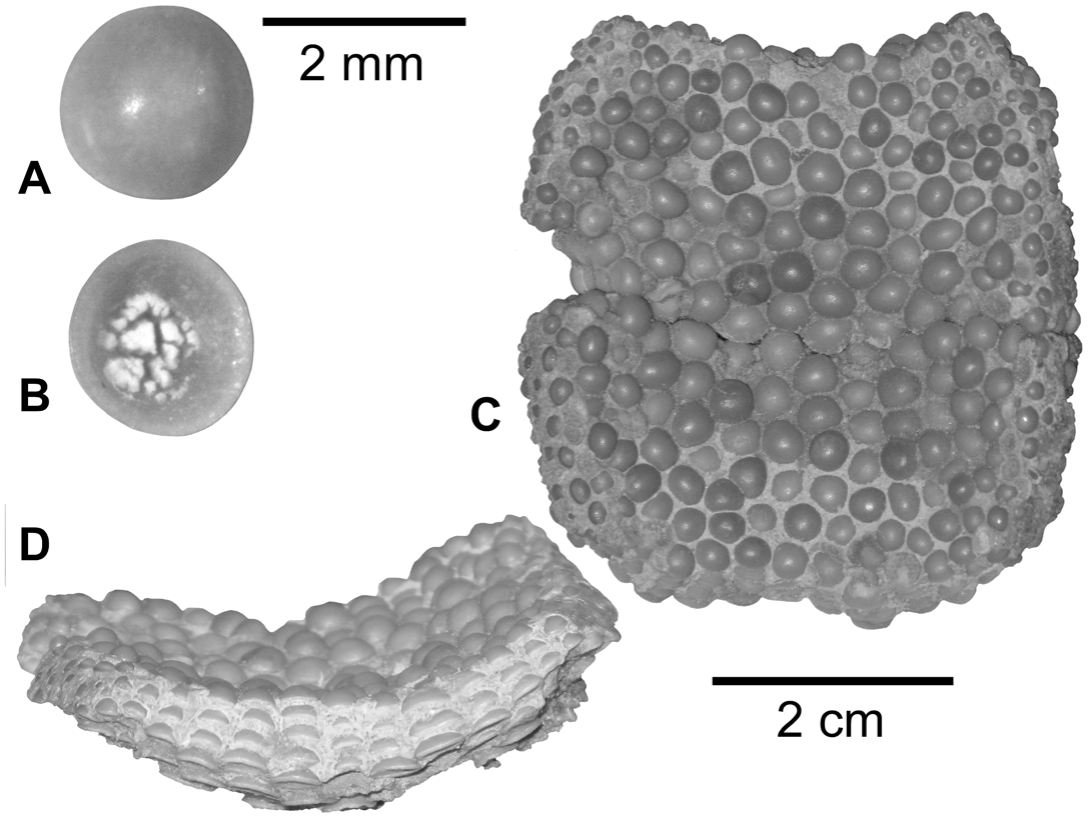
*Egertonia* sp. tooth (A, B) and toothplate (C, D) from the upper Bartonian deposits in Dur At-Talah, Libya (DT-2007-O-1). In, **A**, **C**, occlusal, **B**, near basal, and, **D**, anterior views; C with anterior edge up. [planned for column width].

*EGERTONIA* sp. (Fig 5)

Numerous polished button-like teeth were collected *in situ* in the series sediment (Fig 5A and 5B), and a large phyllodont tooth patch with similar teeth attached was found in the loose arena (Fig 4C and 4D). The teeth have a hemispheric regular crown with a diameter of 1.5–2 mm (Fig 5A). The crown surface is smooth and polished (Fig 5A). The tooth basis has a rounded outline (Fig 5B) with a thin ventral edge. On the tooth patch, all the teeth belong to a same size range (less than one to half ratio), and smaller teeth are located laterally. Functional and replacement teeth are directly stacked in columns of four to five on a bony basal plate. The patch is curved laterally to present a slightly concave occlusal face. This specimen corresponds to a parasphenoid tooth patch, as attested by the presence of anterior surfaces for articulation with the para-ethmoid bones dorsally onto the bony plate.

As summed up by Estes [27], phyllodont tooth patches in fish (i.e., with multiple superposed teeth) occur mostly in the Upper Cretaceous and Paleogene of Europe, Africa and North America. These phyllodont fossils are generally attributed to genera of the extinct family Phyllodontidae (e.g., *Phyllodus*, *Egertonia*, *Pseudoegertonia*, *Paralbula*), which is supposedly related to the elopomorph. Most rarely, Cretaceous and Tertiary phyllodont remains are related to the modern acanthomorph family Labridae (*Labrodon*). Moreover, phyllodont tooth patches are present in extant members of six acanthomorph families, i.e., in the labroid families Labridae, Odacidae and Scaridae, and in the families Sciaenidae, Carangidae and Diodontidae [27]. When compared to the existing Paleogene taxa, as reviewed and described by Estes [27], our phyllodont teeth and tooth patches are consistent with features preserved with *Egertonia*. Notably, the teeth are all smooth, rounded, with a thin base, and stacked in a column in a tooth patch where they all have the same size range.

Order SILURIFORMES

Family CLAROTEIDAE

**Fig 6:**
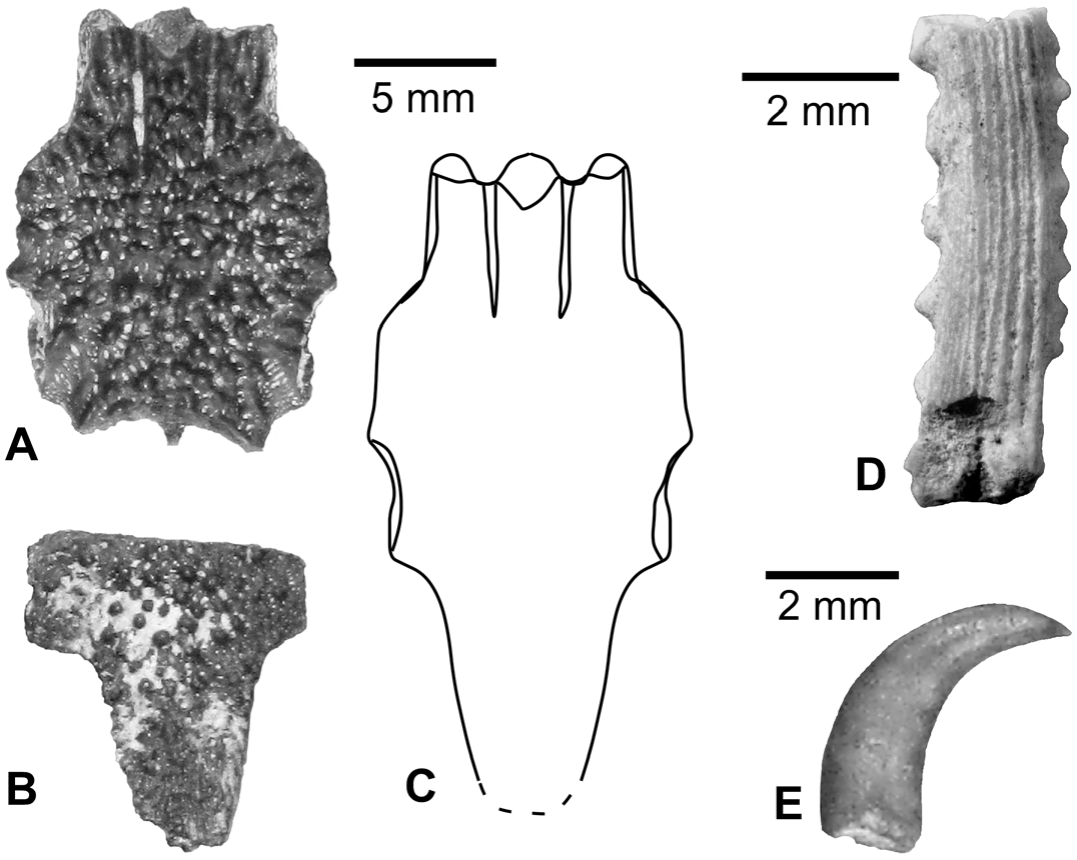
*Chrysichthys/ Clarotes* sp. and other catfish fossils from the upper Bartonian deposits in Dur At-Talah, Libya. **A**–**C**, *Chrysichthys/ Clarotes* sp.: **A**, anterior fragment of a parieto-supraoccipital (DT-2009-O-3), **B**, posterior fragment of a second parieto-supraoccipital (DT-2009-O-3), **C**, composite reconstruction of the parieto-supraoccipital based on **A** and **B**, all in dorsal views; **D**, Siluriformes indet.: fragment of the body of a siluriform pectoral spine with the opening of the median canal (DT-2007-O-1), in dorsal view; **E**, mochokid tooth in lateral view. [planned for column width].

*CHRYSICHTHYS* Bleeker, 1858 [28] or *CLAROTES* Kner, 1855 [29] (Fig 6A–6D)

Two parieto-supraoccipital fragments (Fig 6A and 6B) probably belong to the same species because they are consistent with preserved details, notably the size of the bone (conspecific fossils frequently correspond to specimens with the same size range), the ornamentation, the outline of the bone and its dorso-ventral flatness. They allow reconstruction of the parieto-supraoccipital of this putative species in dorsal view (Fig 6C). It is characterised by the presence of a supraoccipital process with a relatively narrow basis, the lack of any median foramen, and a shape that suggests a connection with four bones on each side, i.e., frontal, sphenotic, pterotic and extrascapular.

The claroteid parieto-supraoccipitals differ from other African catfish by an original combination of characters and by the shape of the posterior process, which have been proposed to distinguish between the *Chrysichthys* and *Clarotes* genera [30]. The parieto-supraoccipital from Dur At-Talah resembles *Chrysichthys* fish rather than *Clarotes* because of the shape and relative dimensions of the supraoccipital process, a feature that classically allows the discrimination of these two modern genera according to Risch [30]. *Chrysichthys* has also been described in sub-contemporaneous deposits from Egypt but on the basis of the shape of scarce dorsal and pectoral spines preserved in their proximal part [6]. However, these current diagnostic features possibly show allometric growth or have been described based on the observation of a few species in each genus; the intra-relationships of the family deserve phylogenetic studies to be better understood. Then, following Otero et al. [10], we suggest that the definition of apomorphies on bony characters and the study of the growth pattern in *Clarotes* and *Chrysichthys* species would possibly help to constrain the phylogeny and systematic palaeontology for the whole subfamily. While *Clarotes* is paucyspecific (two species with only one in the Nilo-Sudan province), *Chrysichthys* is far more diverse and has 33 living species including 12 in the Nilo-Sudan province.

Family MOCHOKIDAE Jordan, 1923 [31]

MOCHOKIDAE indet. (Fig 6E)

In African freshwater, curved teeth are typical of mochokid fish. Within the family, this shape is observed in *Synodontis*, *Microsynodontis*, *Mochokus* and *Chiloglanis* members (Pinton, Otero, pers. obs.). This kind of tooth is usually attributed to *Synodontis* in the fossil record because living species of *Microsynodontis* are very small and because *Chiloglanis* is a genus that inhabits fast moving waters, whereas *Synodontis* are large-bodied and widespread, living in a variety of habitats. However, the description of giant fossil fish in another minute mochokid genus in the late Miocene of Chad, i.e., *Mochokus* [32], suggest that one should not rely on size features to assign fossil remains to a given genus, notably in the case of mochokids. Finally, the identification of mochokids at Dur At-Talah based on a single minute tooth is rather fragile and needs further confirmation.

SILURIFORMES indet.

The spine fragments collected at Dur At-Talah remain indeterminate because they lack the articular head, which alone allows further identification. A few vertebrae recovered are also assigned to catfish.

Order CHARACIFORMES

Family ALESTIDAE

*HYDROCYNUS* Cuvier, 1816 [33]

**Fig 7:**
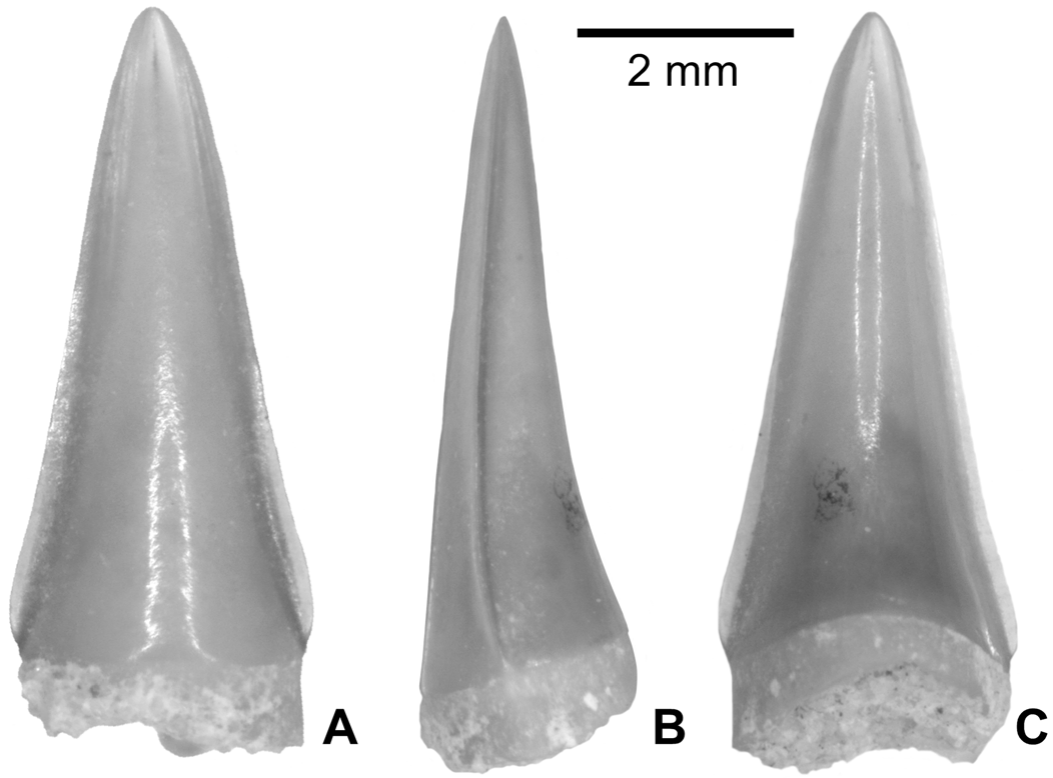
*Hydrocynus* sp. tooth from the upper Bartonian deposits in Dur At-Talah, Libya (DT-2009-O-4). In, **A**, labial, **B**, lateral, and, **C**, lingual views. [planned for column width].

*HYDROCYNUS* sp. (Fig 7)

The *Hydrocynus* teeth collected in Dur At-Talah resemble the modern forms in all of the details that are preserved. They are typically unicuspidate, pointed, with a high labio-lingually compressed crown (Fig 7B) and the labial face is slightly convex (Fig 7A), whereas the lingual one is concave (Fig 7C). On the best-preserved specimens, the cutting edge is marked by a notch at the basis of the crown (Fig 7A and 7C). The enamel-free base is not preserved in the available specimens so that the presence of the characiform festooned base is not observed.

These teeth are similar to extant *Hydrocynus* (the tiger fish) only. The combination of the tooth characters described is only typical of the genus *Hydrocynus* when compared with other African freshwater fish, but also among characiforms in general. Conversely, we are not able to distinguish species on the basis of tooth anatomy. The *Hydrocynus* teeth from Dur At-Talah are, thus, attributed to *Hydrocynus* sp. Finally and despite exact similarity with modern *Hydrocynus* tooth morphology, only a tooth preserved with its festooned base would definitely exclude other assignments.

ALESTIDAE

**Fig 8:**
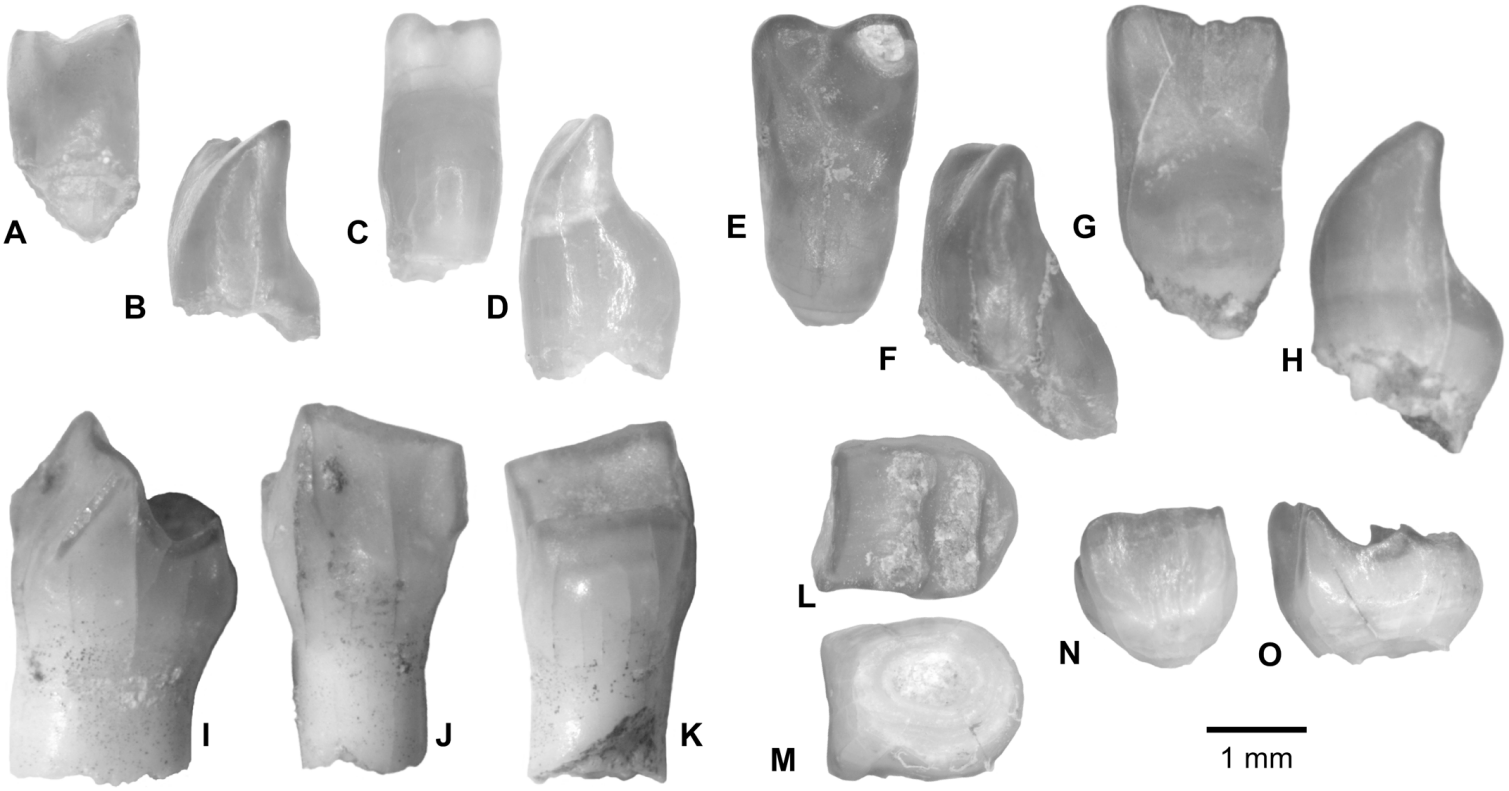
? Characiform multicuspidate teeth from the upper Bartonian deposits in Dur At-Talah, Libya. (**A–H**) four bifidus teeth with an inflated basis [(**A**–**F**) 3 teeth (DT-2009-O-5) and (**G**–**H**) 1 tooth (DT-2009-O-4), in inner (**A**, **C**, **E**, **G**) and lateral (**B**, **D**, **F**, **H**) views], and (**I**–**O**) two multicuspidate teeth (DT-2009-O-4) [first tooth in, **I**, lateral, **J**, outer, and, **K**, inner views; second tooth in, **L**, occlusal, **M**, basal, **N**, outer, and, **O**, lateral views]. [planned for page width].

?CHARACIFORMES indet. and ALESTIDAE indet. (Fig 8)

Today, African characiforms with differentiated dentition show unicuspidate teeth, bicuspidate teeth and molariform to sharp blade teeth. All of these tooth morphologies are found in the subfamily Alestinae (African tetras), whereas the members of the monogeneric Hydrocyninae and Hepsetidae show unicuspidate caniniform teeth, and certain Distichodontidae exhibit typically bilobed teeth. In alestin fish, the various tooth morphologies depend on both the position of the tooth in the jaw bones, especially the premaxillae, and on the taxa. At Dur At-Talah, we have collected bi- and multi-cuspidate teeth. The unicuspidate teeth are significantly smaller (less than one mm) so that our screens are too large mesh to allow their sampling if present. Other alestin-like teeth collected exhibit three different morphologies: one bicuspidate and two molariform.

Bicuspidate teeth with a short shelf (Fig 8A–8H) resemble the teeth of the outer row either of the dentary or of the premaxilla in modern and fossil alestins. Indeed, the teeth of the outer row are less molariform on the premaxilla than on the dentary in some *Alestes*/ *Brycinus* fish, but not in the fossil *Sindacharax* [34,10], and it is also probably not the case in the Dur At-Talah characiform fish (if we accept that they occupy the same position as in alestins). All of the fossil bicuspidate teeth sampled are roughly the same size with less than a factor of 1.5 between the smallest and largest specimens. The cusps are low and rather smooth. They constitute the labial edge of the teeth, which is sometimes slightly crenulated (Fig 8G). The cusps may also show evidence of weathering (Fig 8A). Except for these small variations, the bicuspidate teeth do not vary significantly from each other in their morphology. Finally, as there are only a few variations in these alestin-like outer row teeth depending on the taxa, the bicuspidate teeth from Dur At-Talah may reasonably be interpreted as belonging to several species.

Molariform teeth with minute cusps organised in rows or crests (Fig 8I–8O) may correspond to teeth of the premaxilla inner row when compared to the dentition of ascertained alestins. The higher crest might be located at the lingual face and constitute the cutting edge (Fig 8I and 8O). It is straight in the occlusal view (Fig 8L). Internally, relative to the crest, the tooth extends in a lower shelf with a rounded edge. Depending on the tooth, one or two lower crests or minute cusp rows develop on the shelf, parallel to the lingual main cutting edge. In the occlusal view, the teeth have more squarish outlines and the cusps are much less differentiated (when seen) than in any modern and fossil alestin fish. In fact, they do not resemble any of the teeth described so far in the fossil record nor in the modern record. Variation in their shape and in the number of cusp rows or crests can feature either their position on the jaw bone or different taxa appurtenance. Nevertheless, differences in the shape and in the size (up to a factor of 2 in height and up to a factor of 1.5 in width at the lingual edge) correlate with each other, which suggests that two taxa were present. However, without any articulated jaw, we are not able to defend this assumption.

Based on tooth heterodonty and distribution in fossil and modern alestins (notably in *Alestes/Brycinus* and *Sindacharax*), we assume that bicuspidate and molariform characiform teeth from Dur At-Talah probably belong to at least two taxa that can be distinguished by their molariform tooth morphology.

Fossil multicuspidate teeth are traditionally referred to as characiform fish (e.g., [35]), and in the case of African fossils, the attribution to Alestidae (subfamily Alestinae) appeared reasonable because it includes all of the African characiform fish with molariform teeth. However, while the fossil *Sindacharax* is reasonably related to the extant subfamily Alestinae on the basis of shared morphological traits with *Alestes/Brycinus* members [10], the teeth from Dur At-Talah do not resemble alestin fish teeth enough to further speculate on their position within the family and even to confidentially assume their appurtenance to this taxon. Indeed, the morphologies observed at Dur At-Talah are described for the first time. While waiting for new elements (jaws with their teeth), the unsolved question of their assignment among characiform fish remains, and we refer to them as? Characiformes indet. Indeed, the three types identified might belong to different taxa including at a familiar level.

Order PERCIFORMES

Family CHANNIDAE

*PARACHANNA* Teugels and Daget, 1984 [36]

**Fig 9:**
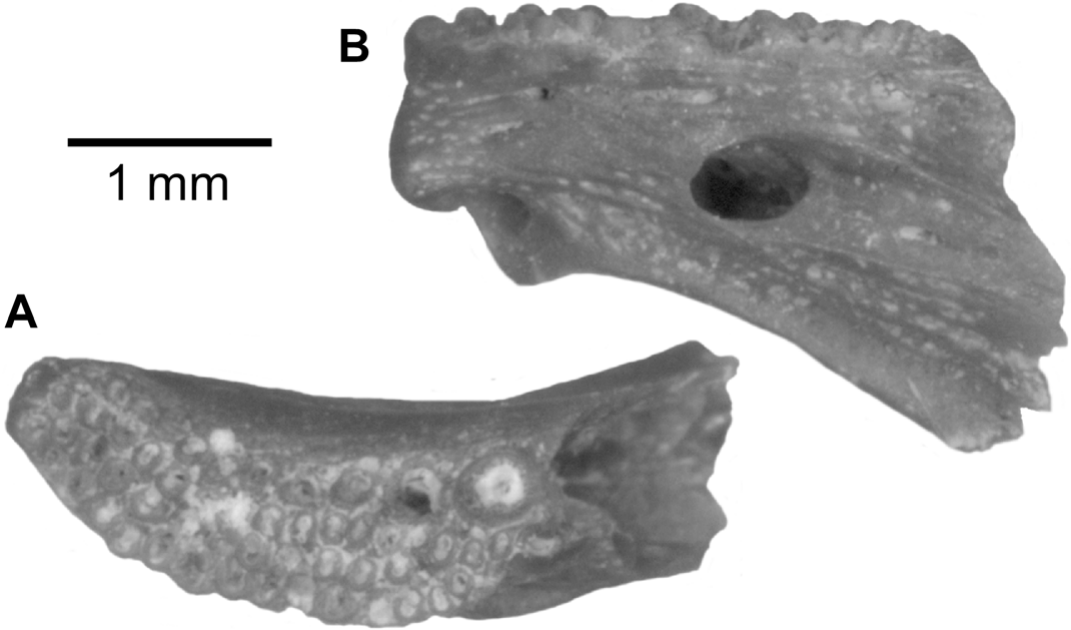
*Parachanna* sp. jaw from the upper Bartonian deposits in Dur At-Talah, Libya. Anterior fragment of a left dentary (DT-2009-O-4), in, **A**, occlusal and, **B**, lateral views. [planned for column width].

*PARACHANNA* sp. (Fig 9)

Two perciform dentaries are attributed to *Parachanna* (Fig 9). They are both preserved in their proximal part and constitute the only material from Dur At-Talah that can be related to this genus. The teeth are not preserved, but the tooth patch exhibits four rows of small tooth sockets anteriorly (Fig 9A). Then, the tooth patch narrows abruptly and only contains two rows: the inner one with tooth sockets that are bigger posteriorly, and an outer row with smaller tooth sockets similar in size to each other (Fig 9A). In lateral view, the dentary exhibits a large pore for the mandibular canal opening on its lateral face and a distinctive symphysal “chin” with a large pore opening ventrally in a notch (Fig 9B).

When compared with other perciforms, notably within African freshwater fish, the two dentaries from Dur At-Talah fit in all the details that are preserved with *Parachanna*, the only extant genus of the family Channidae in Africa. The dentary also resembles in all details with the African fossils of the family: i.e., with *P*. *fayumensensis* from the late Eocene and early Oligocene deposits in El Qatrani, Egypt [37], and with a *Parachanna* sp. dentary collected in the early late Eocene of the Birket Qarun Formation, Egypt [6]. We agree that dentaries do not carry specific characters of *Parachanna* [6], so we refer our isolated dentaries to *Parachanna* sp.

Family CICHLIDAE

*TYLOCHROMIS* Regan, 1920 [38]

**Fig 10:**
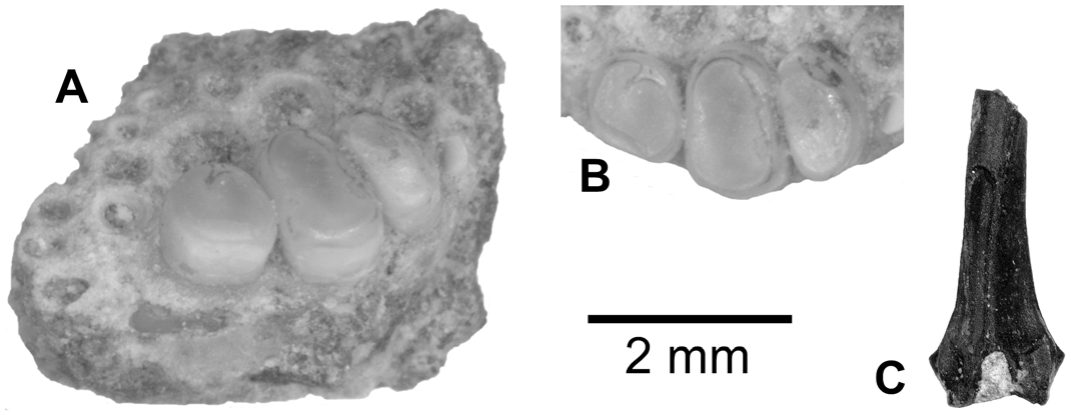
Cichlid remains from the upper Bartonian deposits in Dur At-Talah, Libya. **A**, **B**, left lower pharyngeal jaw fragment (DT-2009-O-4), **A**, in postero-median view, and, **B**, detail of the remaining teeth in occlusal view; **C**, median fin spine in anterior view. [planned for column width].

CICHLIDAE indet. and cf.? *TYLOCHROMIS* sp. (Fig 10)

Two fossils are assigned to cichlid fish, i.e., half a lower pharyngeal jaw with three teeth attached (Fig 10A and 10B) and a spine of a median fin (Fig 10C). The hemi-pharyngeal jaw is broken anteriorly and the right horn is missing (Fig 10A and 10B). The toothed surface of the lower pharyngeal jaw might have been sub-triangular. Along the midline, there are three large flattened teeth and smaller tooth sockets in a longitudinal row (Fig 10A). Lateral to this row, there are parallel sets of small empty sockets that are smaller towards the edge of the jaw (Fig 10A). The surface of the preserved teeth shows no signs of cusps or ridges. These flattish teeth from Dur At-Talah exhibit a completely flattened occlusal area, and the crown edges are straight and vertical. The largest tooth of the pharyngeal jaw is smaller than 2 mm in its main dimension. The median fin spine is very small. It classically shows a slightly asymmetrical body above a head depressed proximally below the median pore. This spine is also characterised by a squarish head, which is higher and less enlarged than in *Lates*. Conversely, the morphology resembles cichlid spines, and more particularly cichlid dorsal fin spines with an articular head that is usually higher than in the anal fin.

The toothed pharyngeal lower jaw collected at Dur At-Talah shows an overall resemblance with certain *Tylochromis* fish, including a fossil from the Lower Oligocene of El Qatrani, which was referred to this genus [39]. The flattish occlusal surface and the distribution and size of the tooth sockets have not been observed in any fish genus other than *Tylochromis*. However, the characters that would ascertain an attribution to this genus are not preserved [notably labroid fish have pharyngeal jaws with a ventral median keel that allows the insertion of the *transversus ventralis* anteriorly onto the lower pharyngeal jaw, and among labroids, most cichlids exhibit an interdigitating suture on the ventral surface between left and right ceratobranchials [40]. The isolated impaired fin spine with a proximal notch below a pore characterises acanthomorph fish only. We attribute this to a cichlid because they resemble these fish when compared with other African acanthomorph fishes.

**Fig 11:**
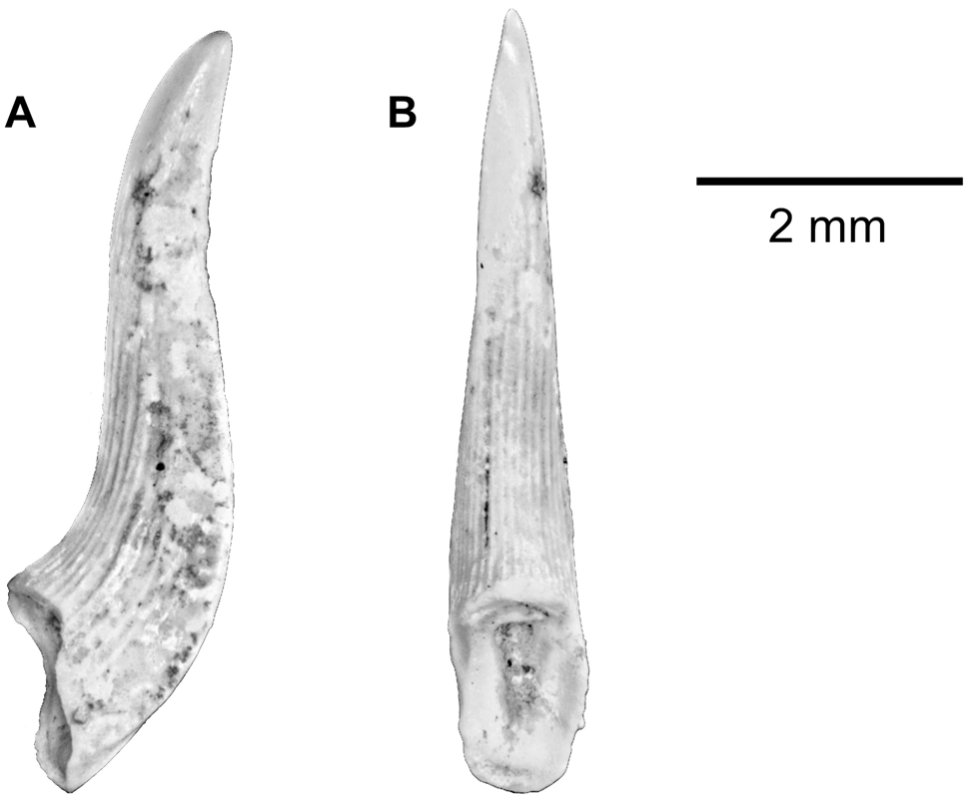
Indeterminate fish tooth from the upper Bartonian deposits in Dur At-Talah, Libya. **A**, lateral, and, **B**, lingual views. [planned for column width].

Order and family indet. (Fig 11)

At least six indeterminate actinopterygian taxa are also present at Dur At Talah because we collected five types of isolated teeth and one type of tooth found attached to pharyngeal bones that remain incertae sedis.

1. Two teeth, one complete but somewhat eroded and one broken, show a morphology that is very different from all other actinopterygian remains (Fig 11). The complete tooth is rather slender with a sigmoid outline in the lateral view. Its base is very concave, with an almost elliptical shape. One can observe the beginning of a pulpar duct, probably present up to the apex of the tooth. There are about 25 folds, mainly distinct at the base of the tooth. Those folds are rounded and some cover three-quarters of the tooth. The lingual part of the tooth is transversely rounded when there is a distinct labial cutting edge from the apex to the base. In labial view, on either side of the cutting edge, the faces are not symmetrical, with one being more convex than the other. The second tooth, broken but better preserved than the first one, allows a better observation of folds, which are very distinct and sharp, sometimes with additional short folds at the base between the main ones. They are mainly developed on the most convex part of the tooth. This first tooth morphology resembles to that of marine fishes like sphyraenids or enchodontids. It is likely that these teeth correspond to marine fishes, the relationships of which are not possible to establish at the moment.
2. Numerous button-like crushing teeth were collected at Dur At-Talah. Most range from globulous to flattish outline and have a larger basal border than *Egertonia*, like in certain other phyllodontid genera.
3. The other button-like teeth have a hemispheric regular crown that is perfectly smooth and polished. They resemble the mormyrid *Hyperopisus* palatine teeth. They are all undetermined as they might also belong to other fish with crushing teeth that frequently inhabit coastal marine environments (e.g., sparid fish). Associated jaws or a tooth patch are required to confidentially attribute these teeth to a particular species.
4. Unicuspidate caniniform teeth are observed in various fishes including some of the taxa that have been previously described. Effectively, a caniniform tooth develops on the alestin lower jaw at the symphysis, as observed in the modern African tetra *Brycinus macrolepidotus*. Caniniform teeth are also present on sparid jaws. For instance, the sun-fish (*Sparus aurata*) shows such teeth at the front of the mouth, whereas crushing flattened teeth cover the jaws at the back of the mouth.
5. A minute labio-lingually bent and flattened bicuspidate tooth was also found at Dur At-Talah. Biscuspidate teeth are found on the jaws of several characiforms (see above) and on the pharyngeal jaws of certain cichlid fish in Africa. However, none of those that we have observed so far correspond to this morphology. Here again, lacking the associated bone, the tooth cannot be definitively referred to a given taxon.
6. Finally, pharyngeal teeth with a pointed crown and a rounded to oval cross-section occur in various teleost fish and their attribution remains uncertain.

## Discussion

### The Middle/Late Eocene African Fish Diversity and the Early Record of Modern Fish Genera

*Protopterus* jaws and *Polypterus* scales and pinnulae were recovered at Dur At-Talah. These genera belong to ancient lineages of vertebrates, respectively dipnoans (sarcopterygians) and cladistians (actinopterygians). In the literature, they are known as being the two oldest genera of the African freshwater ichthyofauna, and for having on this continent a widespread, rich and presumably diverse fossil record, notably north to the Central African Shear Zone (e.g., [3]). More precisely, these two extant genera have been recognised long before the mid-Eocene time, notably in northern Africa (e.g., [3]). Their presence at Dur At-Talah and at Birket Qarun was, thus, expected. On tooth morphology, we recognise *Gymnarchus* sp. and *Hydrocynus* sp., respectively the aba-fish and the tiger fish. The identification of the latter at Dur At-Talah will be definitely confirmed with the discovery in this outcrop of a jaw or at least a tooth preserved with the articulating base, as it is the case in Birket Qarun [6]. In the modern fauna, these genera show low diversity with respectively one and three species extant; and they are each the only known genus in their own family and subfamily, respectively (Gymnarchidae and Alestidae Hydrocyninae). The features exhibited by their teeth are not found in any other extinct or extant fish, and they are clearly different from the teeth of relatives, among osteoglossiforms and characiforms, respectively. Along with Egyptian fossils [6], the material presented constitutes the earliest record of this tooth morphology in Africa. It corresponds to the rise of these two genera in African freshwaters. This is also the case of certain catfish fossils collected at Dur At-Talah and Birket Qarun which are also the earliest record of modern genera. First, the presence of *Chrysichthys*/ *Clarotes* fish is sustained based on diagnostic features observed in the parieto-supraoccipitals collected at Dur At-Talah (this paper), and on the morphology of pectoral and dorsal spines sampled at Birket Qarun [6]. Indeed, Murray and Budney [41] identified a fossil catfish species that they attributed to this extant genus, i.e., *Chrysichthys mahengeensis*, in the middle Eocene of Mahenge (in Tanzanian crater lake deposits, dated between 45 and 46 Ma). However, their generic attribution is based on the lack of common features with other African modern catfish genera and not on the basis of a shared feature with *Chrysichthys* members. They even noted that “the fossil may in fact represent a new genus” and justify their attribution by their wish to stress its relative affinity with *Chrysichthys* ([41]:985). In our opinion, this generic attribution is thus not valid. The Eocene Northern African occurrence of *Chrysichthys* /*Clarotes* from Egypt and Libya are the oldest known so far. Second, the presence of *Auchenoglanis* is based on the morphology of fin spines from Birket Qarun [6]. Besides ascertain members of the family Claroteidae, the African family Mochokidae also seem to have its earlier fossil records now identified in the Eocene of Dur At-Talah (this paper) and Birket Qarun [6]. But the available fossil remains (teeth) do not allow the recognition of a particular genus and they remain indeterminate in their family. Indeed, in this case we can even not exclude that these teeth belong to another fish.

The Eocene freshwater deposits at Dur At-Talah and Birket Qarun also hosted freshwater perciforms. First, a *Lates* was described by Arambourg and Magnier at Gebel Coquin [11]. If their attribution is correct, it is the oldest known species, since the next one in the fossil record is known from Early Oligocene deposits in Egypt [42]. Second, *Tylochromis*, a basal member in the family Cichlidae in Africa [43], possibly has its first record in Dur At-Talah; however, more material is required to ascertain this early occurrence. Third and last, the Afro-Asian family Channidae (snake-head fish) also has an African fossil record beginning in the Eocene outcrops in northern Africa with fossils known from both Libya (this paper) and Egypt [6].

Previously, the Eocene African ichthyofaunal elements in Libya had been determined only through the description of a small assemblage in Dur At-Talah (their Gebel Coquin) by Arambourg and Magnier [11]. The current description of a much richer assemblage from that site is possible due to a new wave of field projects including the systematic use of screen-sampling methods. As is found in younger sites (e.g., [44]), an important part of the fish diversity might be revealed by small elements, the diagnostic potential of which depends on the taxon. It is particularly good for fish with differentiated teeth and fragile bones. With the exception of the archaic *Protopterus* and *Polypterus*, all of the taxa identified at a generic level correspond to the earliest record of a modern genus, but we lack the extinct genera that are common later than the Eocene (in the Neogene) in the African freshwater outcrops, such as the characiform *Sindacharax* and the acanthomorph *Semlikiichthys*. The presence of snake-head fish in both Dur At-Talah and Birket Qarun highlights the Asian stamp to the ichthyofauna that has been noticed for Eocene mammals from northern Africa (e.g., [45]).

### Paleobiogeographical and Paleoenvironmental Implications

The New Idam Unit provided the freshwater fish fossil remains and most of the vertebrate remains from Dur At-Talah. This Unit corresponds to a tide-dominated depositional environment. The sediments are extensively bioturbated in the upper part and the associated ichnofacies typically correspond to shallow marine environments (Fig 1). Root and desiccation crack marks testify phases of emersion. Finally, the paleontological content as a whole resembles that from sub-contemporaneous series in Egyptian deposits from the Fayum area [15,16,46,45,7]. When we focus on the freshwater fish, the resemblance between the assemblage from Libya at Dur At-Talah and that from Birket Qarun is striking. Two genera (*Auchenoglanis* and *Alestes/Brycinus*) present in Egypt are lacking in the Libyan assemblage, and all the fishes present at Dur At-Talah are present from the Egyptian sample, including those assigned to? Characiformes (Alison Murray, pers. com. 2015). The difference is even weaker when we consider the abundance of these fossils when present. Notably, the occurrence of *Alestes/Brycinus* at Birket Qarun is supported by a single very minute tooth, so that further screening and systematic fish sampling at Dur At-Talah might reduce the difference. Conversely, we have to recall that the overall resemblance is observed at a generic level. Thus, we suspect that a specific level of identification might reveal hidden differences between the faunas of the two basins. We even cannot exclude a certain provincialism of the ichthyofaunas, as yet suggested by the case of *Protopterus* for which we suspect that a different species inhabits each of the two basins. Nevertheless, the most relevant information provided by their study is that the common Eocene fish content in these two localities at the northern edge of the African continent prefigures for the first time in the fossil record the modern African freshwater ichthyofauna at a regional scale.

With the exception of the phyllodont fish, most of the fossils described in this paper are freshwater fish. However, some of the isolated unidentified teeth might also belong to marine coastal and estuarine taxa, and, overall, numerous marine chondrichthyanss were collected at Dur At-Talah and will be the subject of another paper. With the presence of lungfish, catfish and polypteriforms, the freshwater ichthyofaunas show tropical characteristics between 39 and 37 Ma in the outcrops of Dur At-Talah in Libya (this paper) and Birket Qarun in Egypt [6]. The ichthyological diversity registered confirms the presence of a permanent freshwater environment in the African northern region with a probable hydrological system of significant size and including streams and possibly lakes or ponds (see also environmental discussion on the subcontemporaneous site at Birket Qarun [6]). The outcrops are located at the edge between marine and continental environments in the deltaic domain where alternate dominance by sea eustatic high level and freshwater floods have influenced the Libyan assemblage.
